# Advances in Artificial Intelligence-Assisted Coronary Computed Tomographic Angiography for Atherosclerotic Plaque Characterization

**DOI:** 10.31083/j.rcm2501027

**Published:** 2024-01-15

**Authors:** Qian Chen, Fan Zhou, Guanghui Xie, Chun Xiang Tang, Xiaofei Gao, Yamei Zhang, Xindao Yin, Hui Xu, Long Jiang Zhang

**Affiliations:** ^1^Department of Radiology, Nanjing First Hospital, Nanjing Medical University, 210006 Nanjing, Jiangsu, China; ^2^Department of Radiology, Jinling Hospital, Medical School of Nanjing University, 210002 Nanjing, Jiangsu, China; ^3^Department of Cardiology, Nanjing First Hospital, Nanjing Medical University, 210006 Nanjing, Jiangsu, China

**Keywords:** artificial intelligence, coronary CT angiography, coronary plaque, deep learning

## Abstract

Coronary artery disease is a leading cause of death worldwide. Major adverse cardiac events are associated not only with coronary luminal stenosis but also with atherosclerotic plaque components. Coronary computed tomography angiography (CCTA) enables non-invasive evaluation of atherosclerotic plaque along the entire coronary tree. However, precise and efficient assessment of plaque features on CCTA is still a challenge for physicians in daily practice. Artificial intelligence (AI) refers to algorithms that can simulate intelligent human behavior to improve clinical work efficiency. Recently, cardiovascular imaging has seen remarkable advancements with the use of AI. AI-assisted CCTA has the potential to facilitate the clinical workflow, offer objective and repeatable quantitative results, accelerate the interpretation of reports, and guide subsequent treatment. Several AI algorithms have been developed to provide a comprehensive assessment of atherosclerotic plaques. This review serves to highlight the cutting-edge applications of AI-assisted CCTA in atherosclerosis plaque characterization, including detecting obstructive plaques, assessing plaque volumes and vulnerability, monitoring plaque progression, and providing risk assessment. Finally, this paper discusses the current problems and future directions for implementing AI in real-world clinical settings.

## 1. Introduction

Despite advances in primary and secondary preventive therapy, coronary artery disease (CAD) remains the predominant cause of morbidity and mortality globally [1]. The luminal stenosis of the coronary artery had been thought to be the main variable for risk stratification and the choice of treatment. However, that concept has been challenged during the past decades by the theory that atherosclerotic plaque is more associated with major adverse cardiac events (MACE) rather than obstructive or nonobstructive coronary disease [2, 3]. Previous studies have demonstrated that plaque morphology and composition play a major role in plaque stability and subsequent risk of acute coronary syndrome (ACS) [4, 5].

This renewed interest in atherosclerotic plaque stabilization has resulted in the development of several imaging techniques, including invasive modalities such as intravascular ultrasound (IVUS), optical coherence tomography (OCT), and non-invasive modalities such as computed tomography (CT), magnetic resonance imaging (MRI) and positron emission tomography (PET) [6, 7]. In the recent guidelines, coronary CT angiography (CCTA) has become the first-line diagnostic technique for the management of patients with stable chest pain [8, 9]. CCTA enables the evaluation of the presence, luminal stenosis, composition, and vulnerability of plaques throughout the coronary tree [10, 11]. However, the increasing use of CCTA exams in daily clinical practice has created an enormous challenge for radiologists and clinicians to assess this valuable information on atherosclerotic plaques accurately and efficiently.

Artificial intelligence (AI), as well as related techniques such as radiomics, might be ideally suited to solve these challenges [12, 13, 14]. AI has increasingly been used in CT to assess atherosclerotic plaques and can potentially improve the radiologists’ workflow, reduce image post-processing time, and improve the accuracy of test results [15, 16, 17]. In addition, risk stratification and prognosis can be more accurately evaluated as AI enables the processing of large amounts of data [13]. Several reviews have been published to discuss the AI methods used for CCTA interpretation [17, 18, 19] or plaque characterization [20, 21]. This review focuses on the latest AI applications for characterizing atherosclerotic plaques on CCTA, ranging from detecting luminal stenosis to risk prediction. We performed a search of PubMed, EMBASE, Scopus and Google Scholar for articles published between database inception and June 31, 2023, using the search terms (“coronary CT angiography” OR “coronary computed tomography angiography” OR “cardiac CT”) AND (“artificial intelligence” OR “machine learning” OR “deep learning” OR “computer-aided diagnostic tools” OR “radiomics”) AND (“plaque” OR “atherosclerosis” OR “coronary artery disease” OR “heart” OR “cardiac” OR “cardio” OR “infarct”), with no language restrictions. Recent applications of AI-assisted CT plaque analysis are presented in Table 1 (Ref. [22, 23, 24, 25, 26, 27, 28, 29, 30, 31, 32, 33, 34, 35]). We also highlight existing problems and future directions before the widespread implementation of AI can be adopted in daily clinical practice.

**Table 1.** **Overview of AI applications for plaque analysis in CCTA studies**.

Study	Input data	Methods	Remarks
Detection of plaque stenosis			
Hong *et al*. [22]	156 CCTA scans	CNN	The DL method had a strong correlation with experts for stenosis measurements.
Choi *et al*. [23]	232 CCTA scans	CNN	AI-based software (Cleerly CORONARY, Cleerly Healthcare, New York, USA) showed high diagnostic performance compared with expert readers.
Griffin *et al*. [24]	303 CCTA scans	CNN	AI-based software (Cleerly CORONARY, Cleerly Healthcare, New York, USA) showed high diagnostic performance compared with quantitative coronary angiography.
Liu *et al*. [25]	165 CCTA scans	CNN	AI-based software (CoronaryDoc, ShuKun Techonolgy, Beijing, China) improved radiologists’ diagnostic performance, especially for inexperienced readers.
Quantification of plaque			
Zeleznik *et al*. [26]	1636 CAC scans	CNN	The DL algorithm showed good agreement with expert manual scoring in both ECG-gated and non-gated CT.
Velzen *et al*. [27]	7240 cardiac CT	CNN	The DL algorithm enabled the quantification of coronary and thoracic calcium with good agreement with manual scoring in varied CT protocols and populations.
	and chest CT		
Lin *et al*. [28]	921 CCTA scans	ConvLSTM network	The DL system had excellent agreement compared with expert readers and IVUS for plaque volume measurement.
Jávorszky *et al*. [29]	894 CCTA scans	Attention U-net	The DL algorithm had an excellent agreement with expert plaque segmentation.
Characterization of vulnerable plaque			
Kolossváry *et al*. [30]	25 CCTA scans	Radiomics	Radiomics had superior performance compared to the best conventional CT metrics for the detection of vulnerable plaque.
Al’Aref *et al*. [31]	46 CCTA plaque features in 124 patients	XGBoost	The ML algorithm outperformed a model containing diameter stenosis, lesion length, plaque volume, plaque burden, and HRP to predict culprit lesions.
Lin *et al*. [32]	120 CCTA scans	Radiomics	Radiomics offered incremental value for identifying culprit lesions when added to a model including HRP and plaque volumes.
		Clustering	
Chen *et al*. [33]	299 CCTA scans	Radiomics	Radiomics showed moderate to good diagnostic performance in identifying vulnerable plaques associated with an increased risk of MACE.
		XGBoost	
Prediction of future adverse CAD events			
Motwani *et al*. [34]	25 clinical and 44 CCTA features in 10,030 patients	LogitBoost	The ML model showed better risk prediction value compared with the Framingham risk score, segment stenosis score, or segment involvement score for 5-year all-cause mortality.
Nakanishi *et al*. [35]	46 clinical and 31 CT variables in 66,636 patients	LogitBoost	The ML model outperformed ASCVD risk, segment stenosis score, or segment involvement score in the prediction of both CVD and CHD death.

Notes: AI, artificial intelligence; CCTA, coronary CT angiography; CNN, convolutional neural network; DL, deep learning; CAC, coronary artery calcification; HRP, high-risk plaque; MACE, major adverse cardiac events; ECG, electrocardiogram; IVUS, intravascular ultrasound; ConvLSTM, convolutional long short-term memory; ML, machine learning; ASCVD, atherosclerotic cardiovascular disease; CVD, cardiovascular disease; CHD, congenital heart disease; XGBoost, extreme gradient boosting; CAD, coronary artery disease; CT, computed tomography.

## 2. Terminology and Techniques

**Fig. 1.**
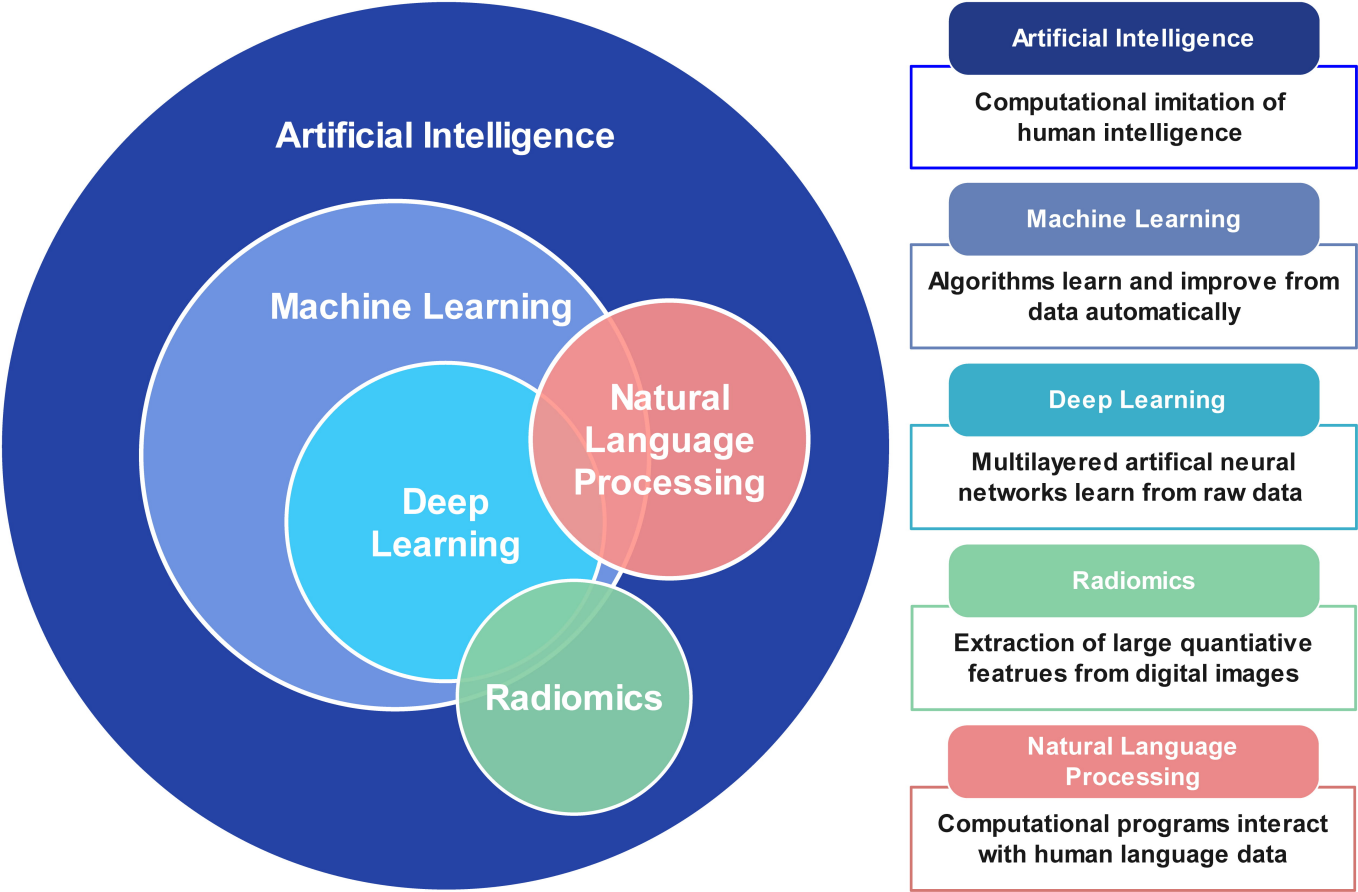
**Basic terminology of artificial intelligence, machine learning, deep learning, and natural language processing**.

AI is a computer science discipline that aims to perform tasks by simulating human intelligence tasks. It contains several subfields, such as machine learning (ML) and deep learning (DL). Fig. 1 summarizes the basic terminology of AI and its relevant techniques.

ML is a branch of AI that involves a growing number of algorithms. ML can be classified into supervised learning, unsupervised learning, and reinforcement learning [36]. Supervised learning, including regression analysis, supports vector machines, and random forest, which refers to learning from labeled data to predict a known outcome. In contrast, unsupervised and reinforcement learning perform unlabeled learning to predict unknown associations. Principal component analysis and clustering are examples of unsupervised learning.

DL is a further subbranch of ML, which uses multilayers of artificial neural networks to automatically learn from raw data [37]. Convolutional neural networks (CNNs) are the most commonly used DL algorithms in medical image analysis. CNNs are composed of 3 major layers: convolution layer, pooling layer, and fully-connected layer. CNNs have been widely used for object detection, classification, and segmentation from images.

Radiomics is the process of extracting high-dimensional quantitative radiological imaging features indiscernible to the human reader [38]. These features characterize the complex spatial relationship between voxels within the region of interest. ML methods can then be combined with radiomics to identify valuable imaging markers to make predictions.

Natural language processing (NLP) methods are advanced AI technologies designed to understand and process human language [39]. In the medical field, NLP can help extract meaningful information from large amounts of data during health care processes, which may reduce clinicians’ workloads and support treatment decisions. Another emerging application of NLP is image analysis [40]. Transformer network is most commonly used in NLP for image processing, such as image detection, segmentation, and reconstruction. NLP can be performed with ML or DL to analyze complex multimodal information.

## 3. Detection of Obstructive Stenosis

Current CCTA reporting is based on the visual estimation of stenosis severity [41]. Accurate assessment of the degree of stenosis is critical to guide treatment decisions by CCTA, including whether to perform invasive coronary angiography. However, there is substantial inter-observer variability among independent readers in real-world practice. A subanalysis of the Prospective Multicenter Imaging Study for Evaluation of Chest Pain (PROMISE) trial demonstrated that expert interpretation at the core laboratory reclassified 41% fewer patients with obstructive plaques (defined as stenosis ≥50%) compared to interpretation at local sites [42]. These results have generated great interest in improving accuracy and reproducibility.

AI-powered techniques have shown promising advances in detecting obstructive CAD. Several AI methods have been developed for automatic coronary artery segmentation and stenosis detection. Kang *et al*. [43] proposed an ML algorithm to automatically detect >25% stenosis in a sample of 42 patients, achieving a high sensitivity of 93% and specificity of 95% compared to expert readers. Additionally, Hong *et al*. [22] trained a CNN to quantify coronary stenosis in 156 patients and found DL measures had an excellent correlation with expert readers (r = 0.957). Recently, the CLARIFY (CT EvaLuation by ARtificial Intelligence For Atherosclerosis, Stenosis and Vascular MorphologY) study analyzed a Food and Drug Administration (FDA)–approved cloud-based software (Cleerly CORONARY, Cleerly Healthcare, New York, USA) to detect obstructive plaques [23]. This study showed that AI-based CCTA had high diagnostic performance in detecting >50% stenosis with a sensitivity and specificity of 80% and 97%, respectively, when compared to results from expert readers. The authors also reported AI and expert readers generated a Coronary Artery Disease Reporting and Data System (CAD-RADS) score that was in agreement within one category in 98.3% of examinations at the per-patient level. The analysis time was about 10 minutes per patient. In a subsequent study by Griffin *et al*. [24], AI-based software (Cleerly CORONARY, Cleerly Healthcare, New York, USA) was evaluated and compared with quantitative invasive angiography in a multicenter cohort of 303 stable patients. The study found that the automated stenosis assessment had sensitivity and specificity of 94% and 68% for detecting obstructive stenosis. However, this software is performed on the cloud, which is a limitation for use in clinical practice. On-site deployment is preferred to meet the demands of various clinical settings, including the management of acute chest pain [44].

## 4. Quantification of Atherosclerotic Plaque

There is increasing evidence demonstrating that the measurement of plaque volume and composition increases the prognostic value of patients who are at higher risk for future CAD events [45, 46, 47]. In a subanalysis of the Scottish Computed Tomography of the HEART (SCOT-HEART) trial, the investigators found that patients with low attenuation plaque (LAP) burden >4% were 5 times more likely to suffer myocardial infarction with a 5-year follow-up [48]. Similarly, the Incident Coronary Syndromes Identified by Computed Tomography (ICONIC) study showed that patients who experienced ACS had a significantly higher burden of LAP compared to those who did not [49]. To date, several semi-automated research software have been developed and shown good concordance of plaque volume and composition compared with gold standard techniques (i.e., IVUS) [50, 51]. However, these semi-automated quantitative plaque analysis software often require substantial manual adjustments, a major hurdle for its routine use in clinical practice [52].

AI algorithms that aim to enhance the automation of quantifying plaques have, therefore, been developed [24, 28, 29]. Liu *et al*. [53] trained a vessel-focused 3D CNN to automatically segment coronary plaques with 25 patients. They found the proposed algorithm achieved dice scores of 0.73, 0.68, and 0.83 for noncalcified plaques, mixed calcified plaques, and calcified plaques, respectively. In a similar study, Jávorszky *et al*. [29] used a 3D U-net model to segment plaques with 308 patients’ 894 CCTA scans. The results showed the DL model had good agreement with expert analysis to quantify total (intra-class correlation [ICC] = 0.88), noncalcified (ICC = 0.84), and calcified (ICC = 0.99) plaque volumes. Recently, Lin *et al*. [28] developed and validated a novel DL algorithm to automatically segment coronary plaques in a multicenter cohort of 921 patients. The DL system showed excellent agreement with IVUS (ICC = 0.95) for total plaque volume measurements at a much shorter analysis time than for expert manual analysis (5.65 ± 1.87 s vs. 25.66 ± 6.79 min per patient). Further, the developed DL-based automated plaque measurements could be used to stratify the risk of CAD events in the SCOT-HEART trial. These studies showed that the DL-based approaches had the potential to reduce the post-processing time with good agreement compared to expert readers. Nevertheless, these AI models are not fully automated solutions for plaque quantification. One or more preprocessing procedures, such as the extraction of coronary artery centerlines, are still needed.

**Fig. 2.**
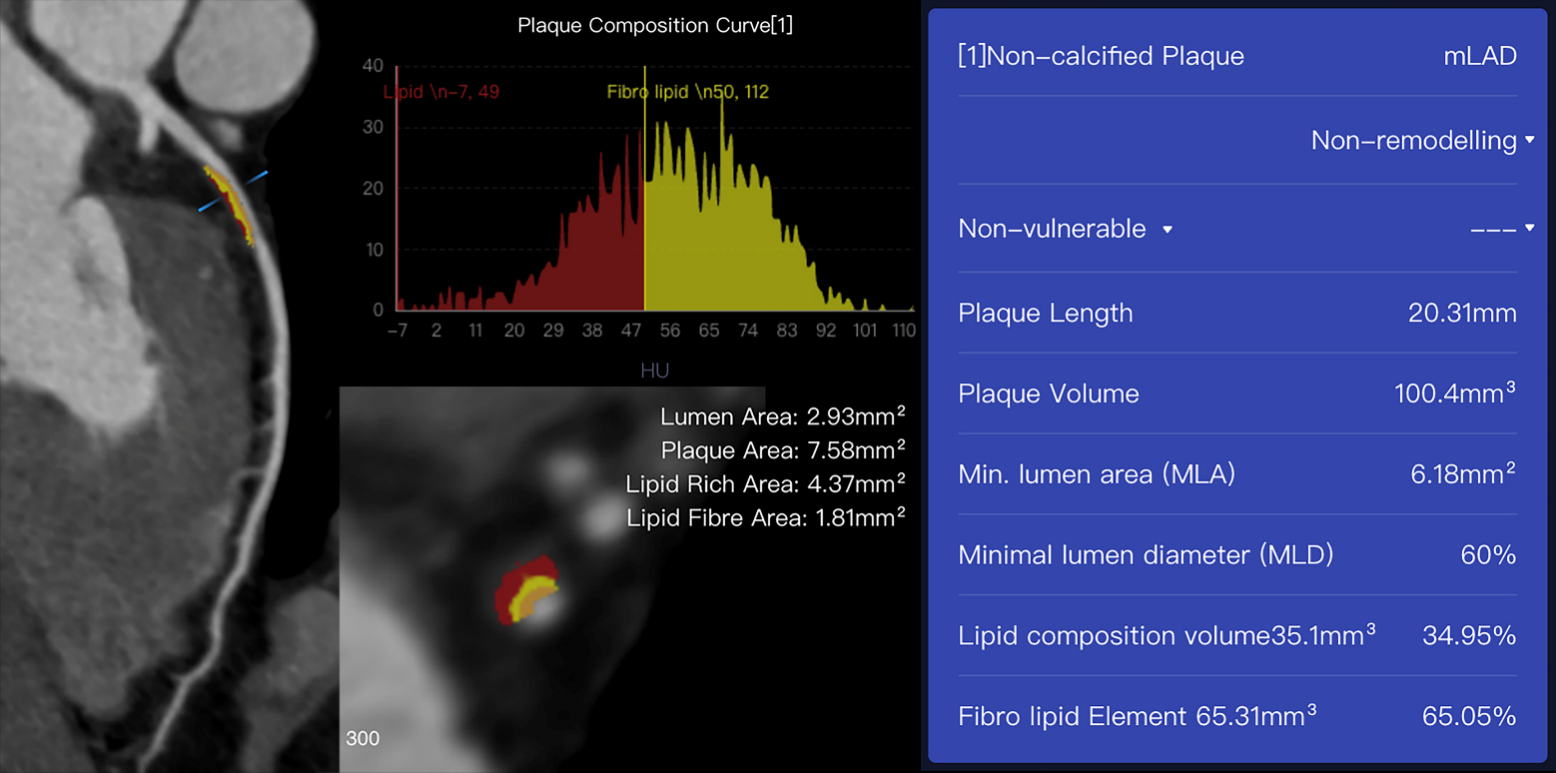
**An example case of plaques assessed using AI-assisted CCTA software (CoronaryDoc, ShuKun Techonolgy, Beijing, China)**. A 41-year-old male with a history of exertional chest pain had a non-calcified plaque with 100.4 ${\mathrm{mm}}^{3}$ in the proximal and middle segments of the left anterior descending coronary artery on CCTA. mLAD, middle segment of left anterior descending artery; CCTA, coronary computed tomography angiography.

Newer quantitative AI-based algorithms are under development, which aims to eliminate manual and labor-intensive processes. Recently, an AI-assisted CCTA plaque software (Cleerly CORONARY, Cleerly Healthcare, New York, USA) has received FDA approval, which uses a series of CNNs to automatically finish coronary artery segmentation, lumen and arterial wall determination, stenosis, and plaque quantification [24]. Initial validation studies have shown that the software had high diagnostic performance in detecting >50% and >70% stenosis compared to expert readers or quantitative invasive angiography. However, a direct comparison of plaque quantification between the AI-assisted software and IVUS has not yet been reported. An example of a fully automatic quantification of an atherosclerotic plaque on CCTA is shown in Fig. 2.

## 5. Characterization of Vulnerable Plaque

Vulnerable plaque rupture is a leading cause of ACS [54]. Histologic and intravascular imaging findings have demonstrated that plaque features associated with culprit lesions at ACS are a large lipid-rich necrotic core, a thin fibrous cap, micro-calcification, positive remodeling, chronic inflammation, and neovascularization [55]. The early identification of vulnerable plaques is important for individualized risk assessment and clinical treatment [6, 56, 57].

CCTA can not only identify both calcified and noncalcified plaques but also characterize specific high-risk plaque features. The major high-risk plaque features include positive remodeling, LAP, the napkin ring sign, and spotty calcification [41]. Multiple studies have shown that the presence of high-risk plaque (HRP) on CCTA was associated with subsequent major adverse cardiovascular events [58, 59, 60]. However, these HRP features are generally assessed visually and only have a modest interobserver agreement, even among expert readers (Kappa = 0.15–0.69) [59, 61]. This highlights the need for improved automated methods for assessing HRP and standardized reporting.

However, studies on the applications of AI for identifying vulnerable plaques are scarce. In a sub-study of the CLARIFY trial, an AI-based software was evaluated to detect HRP features (including positive remodeling and LAP) in 232 patients [62]. Unfortunately, the results showed that AI had poor agreement with 3 expert readers, with a weighted Kappa coefficient of 0.22, 0.17, and 0.26, respectively. To date, automatically quantifying HRP features using AI remains a great challenge.

Radiomics techniques might be feasible to solve these challenges. Several studies have demonstrated that CCTA-based radiomics methods may help detect vulnerable plaques both *in vivo* and *ex vivo * [30, 32, 63]. Kolossváry *et al*. [63] trained a radiomics-based ML model to diagnose histologically verified atherosclerotic lesions on 95 coronary plaques in 7 male donors. The results found that the radiomics model outperformed multiple conventional models (visual assessment, histogram-based assessment, and average Hounsfield unit) in identifying advanced atherosclerotic lesions. In a subsequent study, Kolossváry *et al*. [30] applied CCTA-based radiomics to identify vulnerable plaques in 44 plaques in 25 patients. The study showed that radiomics was superior to conventional CCTA parameters in identifying IVUS-validated attenuated plaques, OCT-defined thin-cap fibroatheroma, and increased ${\mathrm{NaF}}^{18}$-PET uptake lesions (area under the curve [AUC]: 0.72 vs. 0.59, 0.80 vs. 0.66, 0.87 vs. 0.65, all *p* < 0.001). However, these studies involved a relatively small sample size, and the clinical implications of radiomics were not validated. Recently, our team [33] developed a CCTA-derived radiomics model to detect vulnerable plaques defined by IVUS in 299 patients. We found the proposed radiomics model offered incremental value for identifying vulnerable plaques when added to a model including HRP and plaque volumes. We further tested the prognostic value of the radiomics model in an independent prospective cohort of 708 patients over a 3-year follow-up period and found the radiomics model was associated with a higher risk for future adverse cardiac outcomes (adjusted HR, 2.01; *p* = 0.005). However, the extraction of radiomic features in these studies was uniformly followed by semiautomated plaque segmentation. Further development of AI-based automatic software that integrates automated plaque segmentation, radiomics, and ML analyses to identify vulnerable plaques is expected in clinical practice [64].

In addition, coronary inflammation can be measured on CCTA using deep learning methods, and it has been developed as a biomarker named the fat attenuation index (FAI) [65]. The perivascular FAI around culprit lesion precursors increases significantly in patients with ACS compared to non-culprit lesions [66]. FAI was also reported to be associated with ${\mathrm{NaF}}^{18}$ coronary uptake on PET-CT, which is considered the gold standard for mapping plaque inflammation *in vivo* [67]. FAI might be adopted as a new marker for determining vulnerable plaques in the future [68].

## 6. Early Identification and Monitoring of Plaque Progression

It has been thought that most ACS results from the rupture of nonobstructive plaques on angiography months to years before the event [69, 70, 71, 72]. Serial invasive angiography studies showed that nonobstructive lesions can progress rapidly before the acute event occurs [71, 72, 73]. In addition, natural history studies using intravascular imaging also found plaques with high-risk features, including lipid-rich plaques, positive vessel remodeling, and thin-cap fibroatheroma, had a higher probability of progressing [74, 75]. Prior CCTA studies demonstrated that in patients with plaque progression, ACS events are substantially higher compared to patients without progression (14.3% vs. 0.3%, *p* < 0.0001) during 3.9 ± 2.4 years of follow-up [60]. Therefore, it has been proposed that plaque progression may be a necessary step between subclinical atherosclerosis and plaque rupture [76]. These findings suggest that identifying and halting plaque progression may reduce future adverse CAD events.

CCTA enables earlier identification of atherosclerosis and the monitoring of dynamic changes in plaque composition [50]. However, since the composition of each coronary plaque is so diverse, conventional qualitative and quantitative baseline CCTA analysis might be limited in detecting plaques at risk of rapid progression. Han *et al*. [77] applied an ML framework incorporating clinical variables and qualitative and quantitative CCTA plaque features to identify individuals with a higher risk of subsequent plaque progression. The ML model showed higher diagnostic performance in predicting individuals with rapid plaque progression compared to clinical and laboratory models. It should be noted that in this study, plaque progression analysis was based on per-patient level, which pooled the plaque volumes across the major arteries. However, prior studies have shown that the patterns of plaque progression are variable among the major coronary arteries [78]. The identification of specific signatures for predicting plaque progression at a per-lesion level on CCTA is more optimal and has been investigated in many intravascular studies [4, 5, 79]. Novel techniques, such as radiomics, perivascular adipose tissue density, and hemodynamics combined with ML, may provide a better understanding of plaque progression.

Intravascular imaging modalities have been established as the gold standard for assessing the clinically relevant progression of atherosclerosis [80, 81]. Several large clinical trials evaluating the dynamic changes in coronary plaques in response to various treatments have made use of these invasive techniques [82, 83]. However, the target populations in these studies were limited to the inclusion of patients at higher risk. The utility of CCTA in evaluating plaque volume and high-risk features has made it possible to extend its application to monitor the response of various therapies in lower-risk groups.

Several cohort studies and a few randomized controlled trials (RCTs) have been performed to assess temporal changes in plaque volumes with serial CCTA. In a prospective observational study of 121 psoriasis patients, Elnabawi *et al*. [84] found biologic treatment was associated with a significant reduction of noncalcified plaque burden compared to non-biologic treatment at 1-year follow-up CCTA. The international PARADIGM (Progression of Atherosclerotic Plaque Determined by Computed Tomographic Angiography Imaging) trial evaluated the use of statins-induced changes in CCTA-based plaque analysis among 1255 patients [85]. The authors found that statin therapy was related to rapid progression of calcified plaque volume but slower progression of overall plaque volume [85]. Budoff *et al*. [86] conducted an RCT to explore the impact of icosapent ethyl on plaque characterization using CCTA. Eighty patients were randomized to receive either icosapent ethyl or placebo. After 18 months of follow-up, LAP volume was reduced by 17% in the icosapent ethyl group, while the placebo group experienced an increase in LAP volume (109%; *p *= 0.0061) [87]. These data suggest the possibility of applying CCTA in monitoring dynamic changes in plaques over time. With the development of future fully automated plaque analysis software, plaque volumes, and high-risk features could be easily compared between serial scans. CCTA may help to provide more accurate treatment decisions in daily clinical practice.

## 7. Prediction of Future Adverse Cardiac Events

Current CAD prevention guidelines recommend the use of population-based risk calculators, such as the Pooled Cohort Equations and SCORE 2, to estimate a 10-year risk of cardiac events [87, 88]. However, predicting outcomes among patients on an individual level remains challenging.

AI has the potential to provide personalized risk evaluation using the integration of multiple quantitative imaging and clinical variables [89]. Several studies have focused on using AI to predict clinical outcomes in both asymptomatic and symptomatic patients [34, 35, 90]. In a large sample study of 66,636 asymptomatic patients, a comprehensive ML model incorporating 46 clinical variables and 31 non-contrast CT measurements was superior for the prediction of 10-year coronary heart disease and cardiovascular disease deaths (AUC = 0.86) compared to the coronary artery calcium score (AUC = 0.82), atherosclerotic cardiovascular disease (ASCVD) score (AUC = 0.83) and CT variables alone (AUC = 0.83) (all *p <* 0.001) [31]. Similarly, Motwani *et al*. [34] developed an ML-based algorithm to predict 5-year all-cause deaths in 10,030 symptomatic patients. In this study, the ML model containing 25 clinical and 44 CCTA variables showed higher prediction values (AUC = 0.79) compared to the Framingham Risk Score (AUC = 0.61) or CCTA severity scores alone (AUC = 0.64). Individual patient-specific variables on the influence of the results could be explained using AI methods such as Sharpley Additive Explanations (SHAP). The stratification of important clinical variables, laboratory tests, and imaging parameters could potentially guide intensified therapy in high-risk patients or identify novel treatment targets to improve cardiac outcomes. However, the implementation of these advanced AI models in real-world clinical practice remains minimal. Such implementations require linking multi-dimensional datasets, including clinical, imaging, and laboratory data. NLP approaches, particularly transformers, enable dealing with these complex multimodal data [40]. Recently, Zhou *et al*. [91] developed a transformer-based model using unified methods to leverage multimodal information, such as structured or unstructured clinical information and medical images for clinical diagnostics. The result showed the unified model had higher diagnostic performance compared to an image data model and non-unified models for pulmonary disease detection and adverse clinical outcomes prediction of COVID-19. These findings suggest transformer-based models may contribute to facilitating patient care and helping to make clinical decisions.

## 8. Opportunities and Challenges

In cardiovascular imaging, AI algorithms have the potential to improve clinical workflow, accelerate post-processing time, provide objective and accurate results, and guide patient management [13]. Clinically available AI-assisted software for assessing stenosis severity has been shown to be accurate and fast. Integration of fractional flow reserve derived from CCTA (CT-FFR) will further guide the subsequent interventional therapy. Fully automated plaque quantification software is under development, which would expand the potential for CCTA in the evaluation of progression and guide the treatment of atherosclerosis. Radiomics, with automated plaque segmentation, feature extraction, and ML analyses, may increase the diagnostic accuracy and precision of high-risk plaque in routine clinical practice. Finally, personalized AI-based risk scores integrating a vast number of clinical and imaging metrics will be calculated in real time, identifying high-risk patients and providing long-term risk evaluation for future cardiac events.

While AI holds great promise for improving current atherosclerosis imaging, there are several barriers to be addressed and resolved. First, the lack of transparency impedes AI adoption in clinical decision-making. Explanations regarding AI results need to be offered to clinicians and patients. The development of explainable AI approaches is an answer to this limitation [92]. For example, SHAP uses a game theory approach to determine the overall importance of variables and explain how the variables impact individual predictions. Second, many current AI algorithms are developed on small datasets with low variability regarding both image acquisition and patient demographics. These AI algorithms could perform well in the development stage but not generalize well in other datasets. Robust validation with external datasets and prospective, real-world, varied datasets is desired before applying AI tools in clinical practice. Third, adequate evaluation of AI algorithms before adoption in clinical is critical [93]. Despite numerous AI algorithms that have been developed and shown promising outcomes, there are few cardiovascular AI technologies validated in current RCTs [94]. The human-AI interaction and the effect of AI in the radiologic workflow are poorly understood in current clinical studies. An RCT is the best way to determine the actual clinical value of an AI system in which one arm receives AI-assisted care and the other receives usual care [95].

## 9. Conclusions

AI-assisted CCTA has the potential for a comprehensive assessment of atherosclerotic plaque characterization by minimizing human error and saving interpreting time. AI can be also used to provide personalized risk stratification for CAD patients by integrating multiple quantitative imaging and clinical data. However, the explainability, robustness, and generalizability of AI need to be addressed before its wide acceptance in the clinic. Future studies are expected to validate the clinical impact of AI algorithms in daily practice.

## Abbreviations

AI, artificial intelligence; CAD, coronary artery disease; MACE, major adverse cardiac events; ACS, acute coronary syndrome; IVUS, intravascular ultrasound; OCT, optical coherence tomography; CCTA, coronary CT angiography; ML, machine learning; DL, deep learning; CNNs, convolutional neural networks; NLP, natural language processing; LAP, low attenuation plaque; FDA, Food and Drug Administration; ICC, intra-class correlation; HR, hazard ratio; AUC, area under the curve; ASCVD, atherosclerotic cardiovascular disease; ECG, electrocardiogram; CVD, cardiovascular disease; CHD, congenital heart disease.

## References

[1] Roth GA, Mensah GA, Johnson CO, Addolorato G, Ammirati E, Baddour LM (2020). Global Burden of Cardiovascular Diseases and Risk Factors, 1990-2019: Update From the GBD 2019 Study. *Journal of the American College of Cardiology*.

[2] Stone PH, Libby P, Boden WE (2023). Fundamental Pathobiology of Coronary Atherosclerosis and Clinical Implications for Chronic Ischemic Heart Disease Management-The Plaque Hypothesis: A Narrative Review. *JAMA Cardiology*.

[3] Villines TC, Rodriguez Lozano P (2020). Transitioning From Stenosis to Plaque Burden in the Cardiac CT Era: The Changing Risk Paradigm. *Journal of the American College of Cardiology*.

[4] Erlinge D, Maehara A, Ben-Yehuda O, Bøtker HE, Maeng M, Kjøller-Hansen L (2021). Identification of vulnerable plaques and patients by intracoronary near-infrared spectroscopy and ultrasound (PROSPECT II): a prospective natural history study. *Lancet (London, England)*.

[5] Jiang S, Fang C, Xu X, Xing L, Sun S, Peng C (2023). Identification of High-Risk Coronary Lesions by 3-Vessel Optical Coherence Tomography. *Journal of the American College of Cardiology*.

[6] Mintz GS, Matsumura M, Ali Z, Maehara A (2022). Clinical Utility of Intravascular Imaging: Past, Present, and Future. *JACC. Cardiovascular Imaging*.

[7] Mézquita AJV, Biavati F, Falk V, Alkadhi H, Hajhosseiny R, Maurovich-Horvat P (2023). Clinical quantitative coronary artery stenosis and coronary atherosclerosis imaging: a Consensus Statement from the Quantitative Cardiovascular Imaging Study Group. *Nature Reviews. Cardiology*.

[8] Knuuti J, Wijns W, Saraste A, Capodanno D, Barbato E, Funck-Brentano C (2020). 2019 ESC Guidelines for the diagnosis and management of chronic coronary syndromes. *European Heart Journal*.

[9] Gulati M, Levy PD, Mukherjee D, Amsterdam E, Bhatt DL, Writing Committee Members (2021). 2021 AHA/ACC/ASE/CHEST/SAEM/SCCT/SCMR Guideline for the Evaluation and Diagnosis of Chest Pain: Executive Summary: A Report of the American College of Cardiology/American Heart Association Joint Committee on Clinical Practice Guidelines. *Journal of the American College of Cardiology*.

[10] Shaw LJ, Blankstein R, Bax JJ, Ferencik M, Bittencourt MS, Min JK (2021). Society of Cardiovascular Computed Tomography / North American Society of Cardiovascular Imaging - Expert Consensus Document on Coronary CT Imaging of Atherosclerotic Plaque. *Journal of Cardiovascular Computed Tomography*.

[11] Serruys PW, Hara H, Garg S, Kawashima H, Nørgaard BL, Dweck MR (2021). Coronary Computed Tomographic Angiography for Complete Assessment of Coronary Artery Disease: JACC State-of-the-Art Review. *Journal of the American College of Cardiology*.

[12] Sermesant M, Delingette H, Cochet H, Jaïs P, Ayache N (2021). Applications of artificial intelligence in cardiovascular imaging. *Nature Reviews. Cardiology*.

[13] van Assen M, Razavi AC, Whelton SP, De Cecco CN (2023). Artificial intelligence in cardiac imaging: where we are and what we want. *European Heart Journal*.

[14] Antoniades C, Patel P, Antonopoulos AS (2023). Using artificial intelligence to study atherosclerosis, predict risk and guide treatments in clinical practice. *European Heart Journal*.

[15] Alalawi L, Budoff MJ (2022). Recent Advances in Coronary Computed Tomography Angiogram: The Ultimate Tool for Coronary Artery Disease. *Current Atherosclerosis Reports*.

[16] Miceli G, Rizzo G, Basso MG, Cocciola E, Pennacchio AR, Pintus C (2023). Artificial intelligence in symptomatic carotid plaque detection: a narrative review. *Applied Sciences*.

[17] Lin A, Kolossváry M, Motwani M, Išgum I, Maurovich-Horvat P, Slomka PJ (2021). Artificial intelligence in cardiovascular CT: Current status and future implications. *Journal of Cardiovascular Computed Tomography*.

[18] Gudigar A, Nayak S, Samanth J, Raghavendra U, A J A, Barua PD (2021). Recent Trends in Artificial Intelligence-Assisted Coronary Atherosclerotic Plaque Characterization. *International Journal of Environmental Research and Public Health*.

[19] Baeßler B, Götz M, Antoniades C, Heidenreich JF, Leiner T, Beer M (2023). Artificial intelligence in coronary computed tomography angiography: Demands and solutions from a clinical perspective. *Frontiers in Cardiovascular Medicine*.

[20] Cau R, Flanders A, Mannelli L, Politi C, Faa G, Suri JS (2021). Artificial intelligence in computed tomography plaque characterization: A review. *European Journal of Radiology*.

[21] Covas P, De Guzman E, Barrows I, Bradley AJ, Choi BG, Krepp JM (2022). Artificial Intelligence Advancements in the Cardiovascular Imaging of Coronary Atherosclerosis. *Frontiers in Cardiovascular Medicine*.

[22] Hong Y, Commandeur F, Cadet S, Goeller M, Doris MK, Chen X (2019). Deep learning-based stenosis quantification from coronary CT Angiography. *Proceedings of SPIE–the International Society for Optical Engineering*.

[23] Choi AD, Marques H, Kumar V, Griffin WF, Rahban H, Karlsberg RP (2021). CT Evaluation by Artificial Intelligence for Atherosclerosis, Stenosis and Vascular Morphology (CLARIFY): A Multi-center, international study. *Journal of Cardiovascular Computed Tomography*.

[24] Griffin WF, Choi AD, Riess JS, Marques H, Chang HJ, Choi JH (2023). AI Evaluation of Stenosis on Coronary CTA, Comparison With Quantitative Coronary Angiography and Fractional Flow Reserve: A CREDENCE Trial Substudy. *JACC. Cardiovascular Imaging*.

[25] Liu CY, Tang CX, Zhang XL, Chen S, Xie Y, Zhang XY (2021). Deep learning powered coronary CT angiography for detecting obstructive coronary artery disease: The effect of reader experience, calcification and image quality. *European Journal of Radiology*.

[26] Zeleznik R, Foldyna B, Eslami P, Weiss J, Alexander I, Taron J (2021). Deep convolutional neural networks to predict cardiovascular risk from computed tomography. *Nature Communications*.

[27] van Velzen SGM, Lessmann N, Velthuis BK, Bank IEM, van den Bongard DHJG, Leiner T (2020). Deep Learning for Automatic Calcium Scoring in CT: Validation Using Multiple Cardiac CT and Chest CT Protocols. *Radiology*.

[28] Lin A, Manral N, McElhinney P, Killekar A, Matsumoto H, Kwiecinski J (2022). Deep learning-enabled coronary CT angiography for plaque and stenosis quantification and cardiac risk prediction: an international multicentre study. *The Lancet. Digital Health*.

[29] Jávorszky N, Homonnay B, Gerstenblith G, Bluemke D, Kiss P, Török M (2022). Deep learning-based atherosclerotic coronary plaque segmentation on coronary CT angiography. *European Radiology*.

[30] Kolossváry M, Park J, Bang JI, Zhang J, Lee JM, Paeng JC (2019). Identification of invasive and radionuclide imaging markers of coronary plaque vulnerability using radiomic analysis of coronary computed tomography angiography. *European Heart Journal. Cardiovascular Imaging*.

[31] Al’Aref SJ, Singh G, Choi JW, Xu Z, Maliakal G, van Rosendael AR (2020). A Boosted Ensemble Algorithm for Determination of Plaque Stability in High-Risk Patients on Coronary CTA. *JACC. Cardiovascular Imaging*.

[32] Lin A, Kolossváry M, Cadet S, McElhinney P, Goeller M, Han D (2022). Radiomics-Based Precision Phenotyping Identifies Unstable Coronary Plaques From Computed Tomography Angiography. *JACC. Cardiovascular Imaging*.

[33] Chen Q, Pan T, Wang YN, Schoepf UJ, Bidwell SL, Qiao H (2023). A Coronary CT Angiography Radiomics Model to Identify Vulnerable Plaque and Predict Cardiovascular Events. *Radiology*.

[34] Motwani M, Dey D, Berman DS, Germano G, Achenbach S, Al-Mallah MH (2017). Machine learning for prediction of all-cause mortality in patients with suspected coronary artery disease: a 5-year multicentre prospective registry analysis. *European Heart Journal*.

[35] Nakanishi R, Slomka PJ, Rios R, Betancur J, Blaha MJ, Nasir K (2021). Machine Learning Adds to Clinical and CAC Assessments in Predicting 10-Year CHD and CVD Deaths. *JACC. Cardiovascular Imaging*.

[36] Deo RC (2015). Machine Learning in Medicine. *Circulation*.

[37] Esteva A, Robicquet A, Ramsundar B, Kuleshov V, DePristo M, Chou K (2019). A guide to deep learning in healthcare. *Nature Medicine*.

[38] Gillies RJ, Kinahan PE, Hricak H (2016). Radiomics: Images Are More than Pictures, They Are Data. *Radiology*.

[39] Jiang LY, Liu XC, Nejatian NP, Nasir-Moin M, Wang D, Abidin A (2023). Health system-scale language models are all-purpose prediction engines. *Nature*.

[40] Shamshad F, Khan S, Zamir SW, Khan MH, Hayat M, Khan FS (2023). Transformers in medical imaging: A survey. *Medical Image Analysis*.

[41] Cury RC, Leipsic J, Abbara S, Achenbach S, Berman D, Bittencourt M (2022). CAD-RADS™ 2.0 - 2022 Coronary Artery Disease-Reporting and Data System: An Expert Consensus Document of the Society of Cardiovascular Computed Tomography (SCCT), the American College of Cardiology (ACC), the American College of Radiology (ACR), and the North America Society of Cardiovascular Imaging (NASCI). *JACC. Cardiovascular Imaging*.

[42] Lu MT, Meyersohn NM, Mayrhofer T, Bittner DO, Emami H, Puchner SB (2018). Central Core Laboratory versus Site Interpretation of Coronary CT Angiography: Agreement and Association with Cardiovascular Events in the PROMISE Trial. *Radiology*.

[43] Kang D, Dey D, Slomka PJ, Arsanjani R, Nakazato R, Ko H (2015). Structured learning algorithm for detection of nonobstructive and obstructive coronary plaque lesions from computed tomography angiography. *Journal of Medical Imaging (Bellingham, Wash.)*.

[44] Han D, Liu J, Sun Z, Cui Y, He Y, Yang Z (2020). Deep learning analysis in coronary computed tomographic angiography imaging for the assessment of patients with coronary artery stenosis. *Computer Methods and Programs in Biomedicine*.

[45] Bienstock S, Lin F, Blankstein R, Leipsic J, Cardoso R, Ahmadi A (2023). Advances in Coronary Computed Tomographic Angiographic Imaging of Atherosclerosis for Risk Stratification and Preventive Care. *JACC. Cardiovascular Imaging*.

[46] Versteylen MO, Kietselaer BL, Dagnelie PC, Joosen IA, Dedic A, Raaijmakers RH (2013). Additive value of semiautomated quantification of coronary artery disease using cardiac computed tomographic angiography to predict future acute coronary syndrome. *Journal of the American College of Cardiology*.

[47] Hell MM, Motwani M, Otaki Y, Cadet S, Gransar H, Miranda-Peats R (2017). Quantitative global plaque characteristics from coronary computed tomography angiography for the prediction of future cardiac mortality during long-term follow-up. *European Heart Journal. Cardiovascular Imaging*.

[48] Williams MC, Kwiecinski J, Doris M, McElhinney P, D’Souza MS, Cadet S (2020). Low-Attenuation Noncalcified Plaque on Coronary Computed Tomography Angiography Predicts Myocardial Infarction: Results From the Multicenter SCOT-HEART Trial (Scottish Computed Tomography of the HEART). *Circulation*.

[49] Chang HJ, Lin FY, Lee SE, Andreini D, Bax J, Cademartiri F (2018). Coronary Atherosclerotic Precursors of Acute Coronary Syndromes. *Journal of the American College of Cardiology*.

[50] Matsumoto H, Watanabe S, Kyo E, Tsuji T, Ando Y, Otaki Y (2019). Standardized volumetric plaque quantification and characterization from coronary CT angiography: a head-to-head comparison with invasive intravascular ultrasound. *European Radiology*.

[51] Conte E, Mushtaq S, Pontone G, Li Piani L, Ravagnani P, Galli S (2020). Plaque quantification by coronary computed tomography angiography using intravascular ultrasound as a reference standard: a comparison between standard and last generation computed tomography scanners. *European Heart Journal. Cardiovascular Imaging*.

[52] Williams MC, Earls JP, Hecht H (2022). Quantitative assessment of atherosclerotic plaque, recent progress and current limitations. *Journal of Cardiovascular Computed Tomography*.

[53] Liu J, Jin C, Feng J, Du Y, Lu J, Zhou J, Pop M, Sermesant M, Zhao J, Li S, McLeod K, Young A, Rhode K, Mansi T (2019). A vessel-focused 3D convolutional network for automatic segmentation and classification of coronary artery plaques in cardiac CTA. *Statistical Atlases and Computational Models of the Heart. Atrial Segmentation and LV Quantification Challenges: 9th International Workshop, STACOM 2018, Held in Conjunction with MICCAI 2018, Granada, Spain, September 16, 2018, Revised Selected Papers. 1st edn*.

[54] Bhatt DL, Lopes RD, Harrington RA (2022). Diagnosis and Treatment of Acute Coronary Syndromes: A Review. *JAMA*.

[55] Burke AP, Farb A, Malcom GT, Liang YH, Smialek J, Virmani R (1997). Coronary risk factors and plaque morphology in men with coronary disease who died suddenly. *The New England Journal of Medicine*.

[56] Gaba P, Gersh BJ, Muller J, Narula J, Stone GW (2023). Evolving concepts of the vulnerable atherosclerotic plaque and the vulnerable patient: implications for patient care and future research. *Nature Reviews. Cardiology*.

[57] Dweck MR, Maurovich-Horvat P, Leiner T, Cosyns B, Fayad ZA, Gijsen FJH (2020). Contemporary rationale for non-invasive imaging of adverse coronary plaque features to identify the vulnerable patient: a Position Paper from the European Society of Cardiology Working Group on Atherosclerosis and Vascular Biology and the European Association of Cardiovascular Imaging. *European Heart Journal. Cardiovascular Imaging*.

[58] Williams MC, Moss AJ, Dweck M, Adamson PD, Alam S, Hunter A (2019). Coronary Artery Plaque Characteristics Associated With Adverse Outcomes in the SCOT-HEART Study. *Journal of the American College of Cardiology*.

[59] Ferencik M, Mayrhofer T, Bittner DO, Emami H, Puchner SB, Lu MT (2018). Use of High-Risk Coronary Atherosclerotic Plaque Detection for Risk Stratification of Patients With Stable Chest Pain: A Secondary Analysis of the PROMISE Randomized Clinical Trial. *JAMA Cardiology*.

[60] Motoyama S, Ito H, Sarai M, Kondo T, Kawai H, Nagahara Y (2015). Plaque Characterization by Coronary Computed Tomography Angiography and the Likelihood of Acute Coronary Events in Mid-Term Follow-Up. *Journal of the American College of Cardiology*.

[61] Maroules CD, Hamilton-Craig C, Branch K, Lee J, Cury RC, Maurovich-Horvat P (2018). Coronary artery disease reporting and data system (CAD-RADS^TM^): Inter-observer agreement for assessment categories and modifiers. *Journal of Cardiovascular Computed Tomography*.

[62] Jonas RA, Weerakoon S, Fisher R, Griffin WF, Kumar V, Rahban H (2022). Interobserver variability among expert readers quantifying plaque volume and plaque characteristics on coronary CT angiography: a CLARIFY trial sub-study. *Clinical Imaging*.

[63] Kolossváry M, Karády J, Kikuchi Y, Ivanov A, Schlett CL, Lu MT (2019). Radiomics versus Visual and Histogram-based Assessment to Identify Atheromatous Lesions at Coronary CT Angiography: An ex Vivo Study. *Radiology*.

[64] Motwani M (2022). Are You a Robot?: Please Select the Images Containing Unstable Plaque. *JACC. Cardiovascular Imaging*.

[65] Antonopoulos AS, Sanna F, Sabharwal N, Thomas S, Oikonomou EK, Herdman L (2017). Detecting human coronary inflammation by imaging perivascular fat. *Science Translational Medicine*.

[66] Kuneman JH, van Rosendael SE, van der Bijl P, van Rosendael AR, Kitslaar PH, Reiber JHC (2023). Pericoronary Adipose Tissue Attenuation in Patients With Acute Coronary Syndrome Versus Stable Coronary Artery Disease. *Circulation. Cardiovascular Imaging*.

[67] Kwiecinski J, Dey D, Cadet S, Lee SE, Otaki Y, Huynh PT (2019). Peri-Coronary Adipose Tissue Density Is Associated With ^19^18F-Sodium Fluoride Coronary Uptake in Stable Patients With High-Risk Plaques. *JACC. Cardiovascular Imaging*.

[68] Antoniades C, Tousoulis D, Vavlukis M, Fleming I, Duncker DJ, Eringa E (2023). Perivascular adipose tissue as a source of therapeutic targets and clinical biomarkers. *European Heart Journal*.

[69] Newby DE, Adamson PD, Berry C, Boon NA, Dweck MR, SCOT-HEART Investigators (2018). Coronary CT Angiography and 5-Year Risk of Myocardial Infarction. *The New England Journal of Medicine*.

[70] Douglas PS, Hoffmann U, Patel MR, Mark DB, Al-Khalidi HR, Cavanaugh B (2015). Outcomes of anatomical versus functional testing for coronary artery disease. *The New England Journal of Medicine*.

[71] Ambrose JA, Tannenbaum MA, Alexopoulos D, Hjemdahl-Monsen CE, Leavy J, Weiss M (1988). Angiographic progression of coronary artery disease and the development of myocardial infarction. *Journal of the American College of Cardiology*.

[72] Little WC, Constantinescu M, Applegate RJ, Kutcher MA, Burrows MT, Kahl FR (1988). Can coronary angiography predict the site of a subsequent myocardial infarction in patients with mild-to-moderate coronary artery disease. *Circulation*.

[73] Dacanay S, Kennedy HL, Uretz E, Parrillo JE, Klein LW (1994). Morphological and quantitative angiographic analyses of progression of coronary stenoses. A comparison of Q-wave and non-Q-wave myocardial infarction. *Circulation*.

[74] Yamamoto MH, Yamashita K, Matsumura M, Fujino A, Ishida M, Ebara S (2017). Serial 3-Vessel Optical Coherence Tomography and Intravascular Ultrasound Analysis of Changing Morphologies Associated With Lesion Progression in Patients With Stable Angina Pectoris. *Circulation. Cardiovascular Imaging*.

[75] Araki M, Yonetsu T, Kurihara O, Nakajima A, Lee H, Soeda T (2021). Predictors of Rapid Plaque Progression: An Optical Coherence Tomography Study. *JACC. Cardiovascular Imaging*.

[76] Ahmadi A, Argulian E, Leipsic J, Newby DE, Narula J (2019). From Subclinical Atherosclerosis to Plaque Progression and Acute Coronary Events: JACC State-of-the-Art Review. *Journal of the American College of Cardiology*.

[77] Han D, Kolli KK, Al’Aref SJ, Baskaran L, van Rosendael AR, Gransar H (2020). Machine Learning Framework to Identify Individuals at Risk of Rapid Progression of Coronary Atherosclerosis: From the PARADIGM Registry. *Journal of the American Heart Association*.

[78] Bax AM, Lin FY, van Rosendael AR, Ma X, Lu Y, van den Hoogen IJ (2022). Marked variation in atherosclerotic plaque progression between the major epicardial coronary arteries. *European Heart Journal. Cardiovascular Imaging*.

[79] Waksman R, Di Mario C, Torguson R, Ali ZA, Singh V, Skinner WH (2019). Identification of patients and plaques vulnerable to future coronary events with near-infrared spectroscopy intravascular ultrasound imaging: a prospective, cohort study. *Lancet (London, England)*.

[80] Räber L, Mintz GS, Koskinas KC, Johnson TW, Holm NR, Onuma Y (2018). Clinical use of intracoronary imaging. Part 1: guidance and optimization of coronary interventions. An expert consensus document of the European Association of Percutaneous Cardiovascular Interventions. *European Heart Journal*.

[81] Truesdell AG, Alasnag MA, Kaul P, Rab ST, Riley RF, Young MN (2023). Intravascular Imaging During Percutaneous Coronary Intervention: JACC State-of-the-Art Review. *Journal of the American College of Cardiology*.

[82] Räber L, Ueki Y, Otsuka T, Losdat S, Häner JD, Lonborg J (2022). Effect of Alirocumab Added to High-Intensity Statin Therapy on Coronary Atherosclerosis in Patients With Acute Myocardial Infarction: The PACMAN-AMI Randomized Clinical Trial. *JAMA*.

[83] Nicholls SJ, Puri R, Anderson T, Ballantyne CM, Cho L, Kastelein JJP (2016). Effect of Evolocumab on Progression of Coronary Disease in Statin-Treated Patients: The GLAGOV Randomized Clinical Trial. *JAMA*.

[84] Elnabawi YA, Dey AK, Goyal A, Groenendyk JW, Chung JH, Belur AD (2019). Coronary artery plaque characteristics and treatment with biologic therapy in severe psoriasis: results from a prospective observational study. *Cardiovascular Research*.

[85] Lee SE, Chang HJ, Sung JM, Park HB, Heo R, Rizvi A (2018). Effects of Statins on Coronary Atherosclerotic Plaques: The PARADIGM Study. *JACC. Cardiovascular Imaging*.

[86] Budoff MJ, Bhatt DL, Kinninger A, Lakshmanan S, Muhlestein JB, Le VT (2020). Effect of icosapent ethyl on progression of coronary atherosclerosis in patients with elevated triglycerides on statin therapy: final results of the EVAPORATE trial. *European Heart Journal*.

[87] Arnett DK, Blumenthal RS, Albert MA, Buroker AB, Goldberger ZD, Hahn EJ (2019). 2019 ACC/AHA Guideline on the Primary Prevention of Cardiovascular Disease: A Report of the American College of Cardiology/American Heart Association Task Force on Clinical Practice Guidelines. *Circulation*.

[88] SCORE2 working group and ESC Cardiovascular risk collaboration (2021). SCORE2 risk prediction algorithms: new models to estimate 10-year risk of cardiovascular disease in Europe. *European Heart Journal*.

[89] Javaid A, Zghyer F, Kim C, Spaulding EM, Isakadze N, Ding J (2022). Medicine 2032: The future of cardiovascular disease prevention with machine learning and digital health technology. *American Journal of Preventive Cardiology*.

[90] Tamarappoo BK, Lin A, Commandeur F, McElhinney PA, Cadet S, Goeller M (2021). Machine learning integration of circulating and imaging biomarkers for explainable patient-specific prediction of cardiac events: A prospective study. *Atherosclerosis*.

[91] Zhou HY, Yu Y, Wang C, Zhang S, Gao Y, Pan J (2023). A transformer-based representation-learning model with unified processing of multimodal input for clinical diagnostics. *Nature Biomedical Engineering*.

[92] Salih A, Boscolo Galazzo I, Gkontra P, Lee AM, Lekadir K, Raisi-Estabragh Z (2023). Explainable Artificial Intelligence and Cardiac Imaging: Toward More Interpretable Models. *Circulation. Cardiovascular Imaging*.

[93] Park SH, Han K, Jang HY, Park JE, Lee JG, Kim DW (2023). Methods for Clinical Evaluation of Artificial Intelligence Algorithms for Medical Diagnosis. *Radiology*.

[94] He B, Kwan AC, Cho JH, Yuan N, Pollick C, Shiota T (2023). Blinded, randomized trial of sonographer versus AI cardiac function assessment. *Nature*.

[95] Park SH, Choi JI, Fournier L, Vasey B (2022). Randomized Clinical Trials of Artificial Intelligence in Medicine: Why, When, and How. *Korean Journal of Radiology*.

